# Retrospective Analysis of the Clinical Efficacy of N-Acetylcysteine in the Treatment of Hepatitis B Virus Related Acute-on-Chronic Liver Failure

**DOI:** 10.3389/fmed.2021.724224

**Published:** 2021-08-05

**Authors:** Meng-Lan Wang, Xiu-Jun Yin, Xue-Lian Li, Fa-Da Wang, Jing Zhou, Ya-Chao Tao, Yong-Hong Wang, Dong-Bo Wu, En-Qiang Chen

**Affiliations:** Center of Infectious Diseases, West China Hospital of Sichuan University, Chengdu, China

**Keywords:** N-acetylcysteine, hepatitis B, intrahepatic cholestasis, acute-on-chronic liver failure, clinical efficacy

## Abstract

**Objective:** HBV-related acute-on-chronic liver failure (HBV-ACLF) has a high mortality due to severe intrahepatic cholestasis and coagulation dysfunction, thus new treatment measures are urgently needed to improve the therapeutic effect. This study aimed to observe the efficacy of N-acetylcysteine (NAC) in the treatment of HBV-ACLF.

**Methods:** The data of patients with HBV-ACLF admitted to West China Hospital from October 2019 to August 2020 were collected retrospectively, and they were divided into treatment group and control group according to whether they had received additional NAC treatment. The improvement of biochemistry, coagulation function and disease severity score after 14 days of hospitalization were analyzed between two groups.

**Results:** A total of 90 HBV-ACLF patients were included, including 42 patients in treatment group and 48 patients in control group. Compared with baseline, serum TBil, DBil, TBA, GGT and ALP in two groups both decreased significantly, while PTA increased significantly. Interesting, the decrease of serum TBil, DBil and TBA and the increase of PTA in treatment group were all significantly than these in control group. Additionally, more patients in treatment group than control group changed from CTP grade C to grade B. Subgroup analysis of CTP grade C patients showed that the decrease of serum TBil, DBil and TBA and the increase of PTA in treatment group were significantly than these in control group.

**Conclusion:** The NAC treatment may help to improve intrahepatic cholestasis and coagulation dysfunction of HBV-ACLF.

## Introduction

Acute-on-chronic liver failure (ACLF) is a common syndrome, which occurs simultaneously with organ failure and has a high mortality (1). In China, nearly 50,000 people die of liver failure every year, and more than 80% of them are caused by hepatitis B virus (HBV) chronic infection (2). Severe intrahepatic cholestasis and deterioration of coagulation function are not only important clinical features of HBV-related ACLF (HBV-ACLF), but also closely related to the prognosis of patients (1, 3). Reducing intrahepatic cholestasis and promoting the recovery of coagulation function is the key to the comprehensive treatment of HBV-ACLF (4, 5). Thus, how to promote bile excretion and quickly reduce intrahepatic cholestasis has attracted much clinical attention.

Adenosylmethionine and ursodeoxycholic acid are two important drugs in the treatment of intrahepatic cholestasis (6, 7), which can effectively improve the progress of the disease to a certain extent. However, they are not satisfactory in improving the severe intrahepatic cholestasis of HBV-ACLF, so clinicians are constantly looking for new treatments to improve this dilemma (3, 8). For example, artificial liver support therapy is used by some clinicians to try to treat severe cholestatic jaundice, but the clinical effectiveness and cost-effectiveness are not satisfactory (9). Therefore, in addition to liver transplantation and stem cell therapy (10, 11), clinicians are eager for new drugs and measures to treat intrahepatic cholestasis in HBV-ACLF patients.

Acetylcysteine (NAC) is a small single peptide molecule, which is composed of L-cysteine and acetyl group and contains sulfhydryl group. At present, it is believed that the protective and therapeutic effects of GSH on liver may be related to the maintenance and increase of GSH content in hepatocytes (12). In addition, NAC has the effects of scavenging free radicals and antioxidation, which helps to protect mitochondrial function, inhibit inflammation, and improve liver hemodynamics and microcirculation, thus promoting the repair of hepatocytes (12–14). Therefore, NAC is more and more used in the comprehensive treatment of live failure (15, 16). In this study, we will retrospectively analyze the clinical data of patients with HBV-ACLF treated with NAC to evaluate whether the addition of NAC on the basis of existing comprehensive treatment can further improve the prognosis of patients with HBV-ACLF.

## Patients and Methods

### Study Design and Participants

This is a single center retrospective clinical study, which was carried out in West China Hospital of Sichuan University, with the purpose of evaluating the efficacy and safety of NAC for the treatment of patients with HBV-ACLF. The diagnostic criteria of HBV-ACLF were in accordance with consensus recommendations of the Asian Pacific association for the study of the liver (APASL) (17). This study was conformed strictly to the ethics guidelines of the 1975 Declaration of Helsinki, and approved by the ethics committee of West China Hospital of Sichuan University.

Patients with the following characteristics were deemed eligible for enrollment in this study: (a) symptoms of weak, anorexia, abdominal distension, nausea and other serious gastrointestinal symptoms; (b) laboratory evidences of serum total bilirubin (TBil) ≥10 × ULN μmol/L and international normalized ratio (INR) ≥1.5 or prothrombin activity <40%; (c) positive serum hepatitis B surface antigen (HBsAg) for more than 6 months and detectable serum HBV-DNA; (d) aged 18–65 years, regardless of gender. Patients with any of the following conditions were excluded: (a) serious complications in previous 3 months (e.g., gastrointestinal bleeding, serious infection such as sepsis); (b) other causes of active liver disease, including autoimmune liver diseases, drug-induced liver damage, alcoholic liver disease, genetic metabolic liver disease; (c) evidences of liver cancer or other malignant tumors, severe diabetes and autoimmune diseases; (d) coinfection with hepatitis A, C, D or E viruses, and/or human immunodeficiency virus; (e) important organ dysfunctions not due to liver disease; (f) pregnancy and lactation; (g) receiving artificial liver support treatment; (h) failed to receive comprehensive support treatments.

The patients in treatment group and control group were both received active comprehensive supportive treatment, including glycyrrhizic acid preparation, reduced glutathione, polyene phosphatidylcholine, adenomethionine, ursodeoxycholic acid, human albumin and plasma infusion. Patients in treatment group also received NAC treatment (produced by Hangzhou Minsheng Pharmaceutical Group Co., Ltd), with a dosage of 8 g diluted with 250 mL of 10% glucose (intravenous infusion, once a day) and the course of treatment was not <2 weeks.

### Data Collection and Observation Indicators

In this study, we collected the detailed demographic data of patients (including age, gender, long course of disease, treatment before admission, etc.), and the auxiliary examination results (including blood routine, various biochemical indexes, coagulation function, blood ammonia, blood pressure, etc.) at the time of admission and during hospitalization HBV related virological indicators and various imaging examination reports, as well as adverse reactions possibly related to N-acetylcysteine reported during treatment. In this study, the blood parameter, serum biochemical and electrolyte indices were detected by automated blood cell Analyzer, automated coagulation analyzer and automatic biochemical analyzer (Olympus AU5400, Olympus Corporation, Tokyo, Japan) using the standard procedures. HBV serological markers were evaluated by electrochemiluminescence immunoassay (Elecsys; Roche Diagnostics, China).

In this study, we mainly explore whether there are significant differences between the treatment group and the control group in the indicators related to intrahepatic cholestasis (such as bilirubin, total bile acid, glutamyltranspeptidase and alkaline phosphatase), as well as prothrombin time, albumin, creatinine, MELD and CTP scores.

### Statistical Analysis

Clinical and biochemical data were expressed as frequencies or median/range, as appropriate. Frequencies were compared using the Chi-square test, and the quantitative data was compared using Student's *t*-test (when values were normally distributed) or the nonparametric Mann-Whitney *U*-test. All data were processed by SPSS 18.0 software (SPSS Inc., Chicago, IL, USA) and a value of *P* < 0.05 was considered statistically significant.

## Results

**Figure 1:**
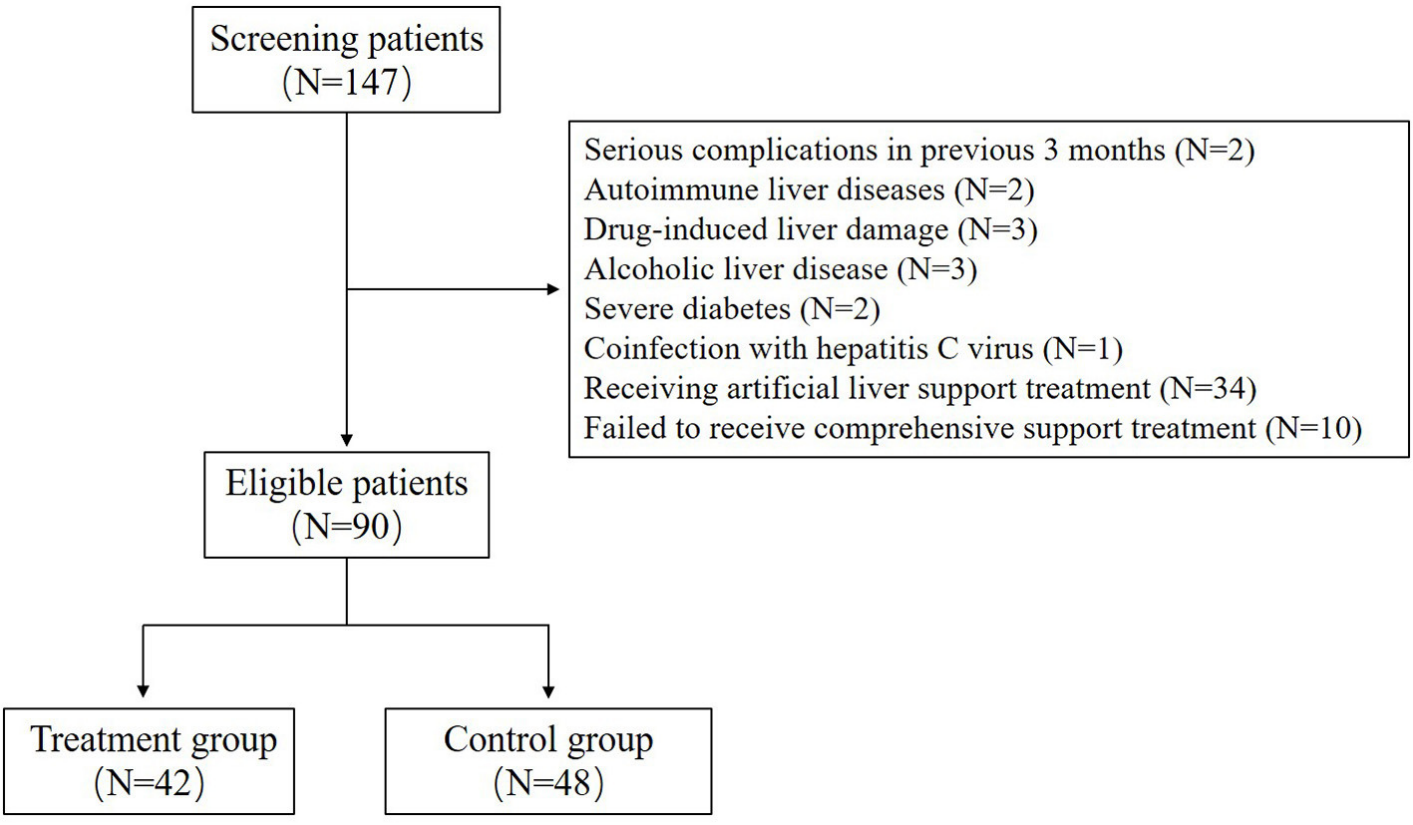
The patient screening and enrollment flow chart.

A total of 90 patients with HBV-ACLF were included in this study, including 42 patients in the treatment group and 48 patients in the control group (Figure 1). In this study, all patients in the two groups had cirrhosis, which was indicated by upper abdominal ultrasound or CT. There was no significant difference in age, gender, duration of disease and antiviral treatment before admission between the two groups; There was no significant difference in HBeAg positive rate and HBV DNA level between the two groups. There was also no significant difference in serum TBil, TBA, ALP and PT between the two groups. In addition, nearly half of the patients in each group had ascites. The detailed information is shown in Table 1.

**Table 1:** Comparison of demographic characteristics and laboratory variables between treatment group and control group.

	**Control (*N* = 48)**	**Treatment (*N* = 42)**	***p*-value**
Age (median, IQR), year	46 (36–53)	44 (38–52)	0.962
Male gender (*n*, %)	27 (56.3)	24 (57.1)	0.932
Duration of disease before admission (median, IQR), day	10.0 (8.0–14.5)	12.0 (9.0–15.0)	0.234
Antiviral therapy before admission			–
Untreated/ Discontinue treatment (*n*, %)	27 (56.2)/21 (43.8)	21 (50.0)/21 (50.0)	0.553
Drug withdrawal time^#^ (median, IQR), month	17.0 (12.0–22.0)	16.0 (13.0–19.0)	0.659
Serum HBVDNA (median, IQR), lg10 IU/mL	4.97 (4.16–6.65)	4.82 (4.00–5.81)	0.125
Serum HBeAg positivity (*n*, %)	22 (45.8)	18 (42.9)	0.777
Serum HBsAg (median, IQR), lg10 IU/mL	3.5 (3.2–3.9)	3.5 (3.3–3.9)	0.836
Alanine aminotransferase (median, IQR), IU/L	999.0 (878.5–1787.5)	1,128 (947–2,027)	0.194
Total bilirubin (median, IQR), μmol/L	398.0 (365.0–438.0)	397.5 (364.0–456.0)	0.372
Direct bilirubin (median,IQR), μmol/L	301.0 (279.0–357.5)	298.0 (276.0–324.0)	0.562
Total bile aci (median, IQR), μmol/L	265.5 (216.0–304.5)	237.5 (192.0–307.0)	0.170
Albumin (median, IQR)	33.4 (30.5–34.7)	32.0 (30.1–35.3)	0.635
Alkaline phosphatase	193.0 (164.5–237.5)	185.0 (145.0–221.0)	0.385
γ-glutamyltranspeptidase	253.0 (205.5–299.5)	295.0 (237.0–355.0)	0.009
Blood ammonia	80.3 (61.8–94.4)	63.9 (45.3–89.5)	0.033
Serum creatinine	84.0 (75.5–93.5)	83.0 (66.0–102.0)	0.534
Alpha fetoprotein	37.5 (18.7–72.0)	28.7 (15.8–46.2)	0.251
Prothrombin time	30.5 (28.7–36.4)	32.3 (28.9–37.4)	0.275
International Normalized Ratio	2.6 (2.4–3.1)	2.7 (2.4–3.2)	0.232
Blood sodium	140.0 (136.5–142.5)	138.5 (135.0–142.0)	0.135
Hepatic encephalopathy (*n*, %)	6 (12.5)	5 (11.9)	0.931
Spontaneous Bacterial Peritonitis (*n*, %)	18 (37.5)	14 (33.3)	0.680
Ascites (*n*, %)	23 (47.9)	20 (47.6)	0.978
Hepatorenal syndrome (*n*, %)	3 (6.3)	5 (11.9)	0.465
MELD score (median, IQR)	28.0 (26.0–31.0)	29.0 (26.0–32.0)	0.586
Child-Turcotte-Pugh classification: A/B/C (*n*, %)	0 (0.0)/9 (18.8)/39 (81.3)	0 (0.0)/13 (31.0)/29 (69.0)	0.179

*Note: The symbol ^#^ represents the time when the patient has stopped taking antiviral drugs*.

**Figure 2:**
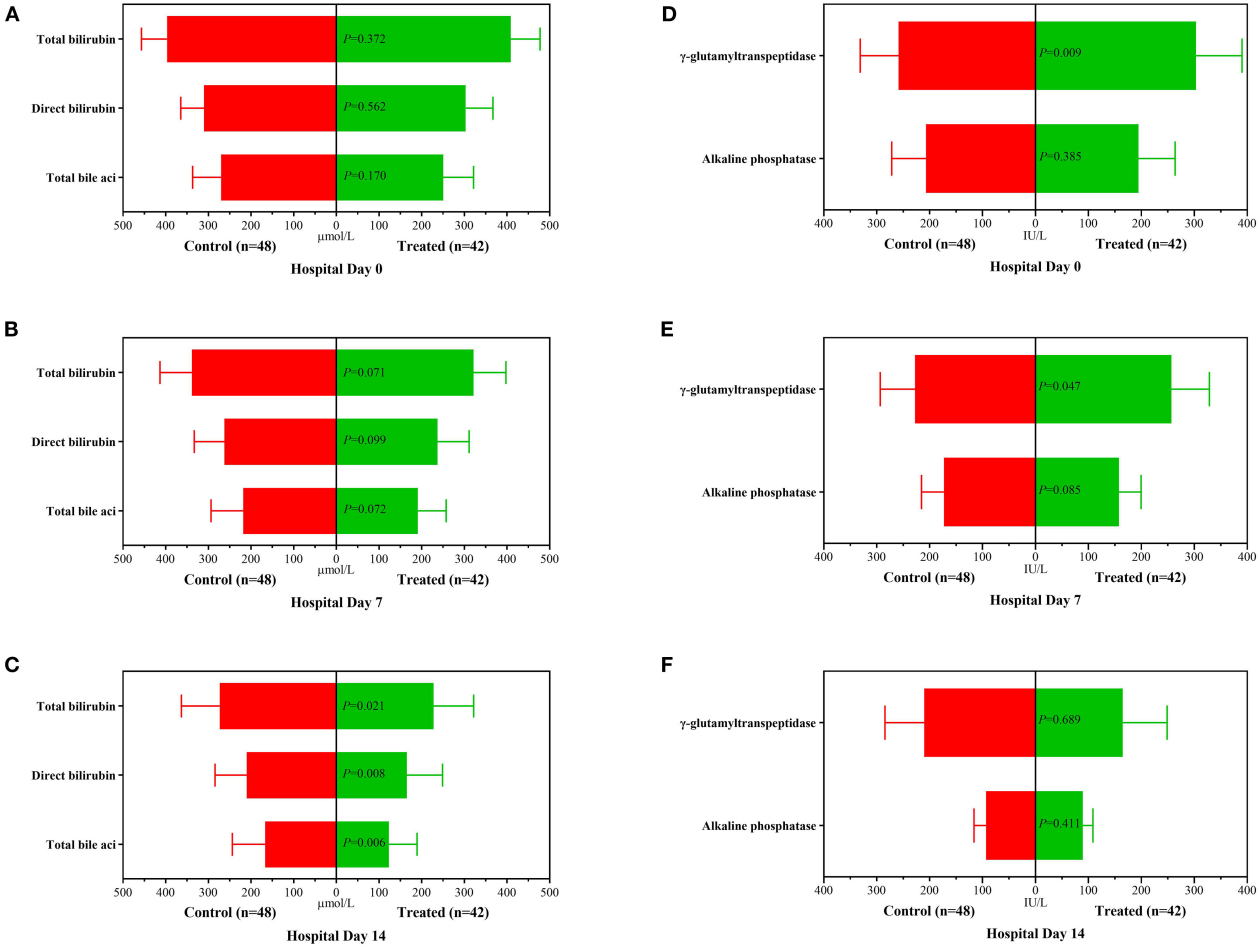
Changes of biochemical variables before treatment, 1 and 2 weeks after treatment between the treatment group and the control group. **(A)**: Distribution of serum TBil, DBil and TBA before treatment; **(B)**: Distribution of serum TBil, DBil and TBA after 1 week of treatment; **(C)**: Distribution of serum TBil, DBil and TBA after 2 weeks of treatment; **(D)**: Distribution of serum ALP and GGT before treatment; **(E)**: Distribution of serum ALP and GGT after 1 week of treatment; **(F)**: Distribution of serum ALP and GGT after 2 weeks of treatment.

**Figure 3:**
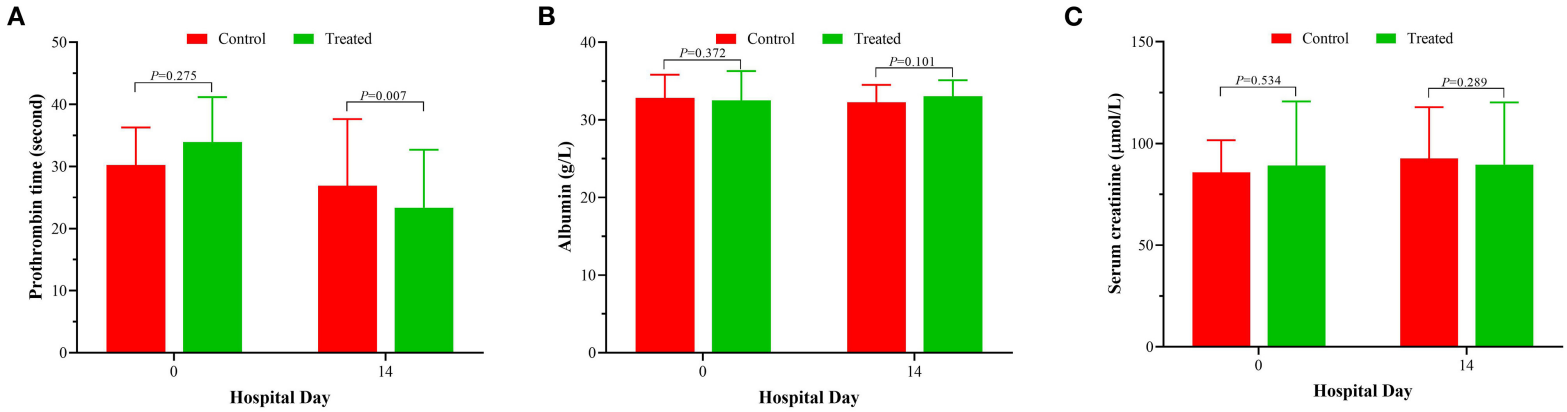
Changes of prothrombin time **(A)**, albumin **(B)** and serum creatinine **(C)** after 2 weeks of treatment between the treatment group and the control group.

**Figure 4:**
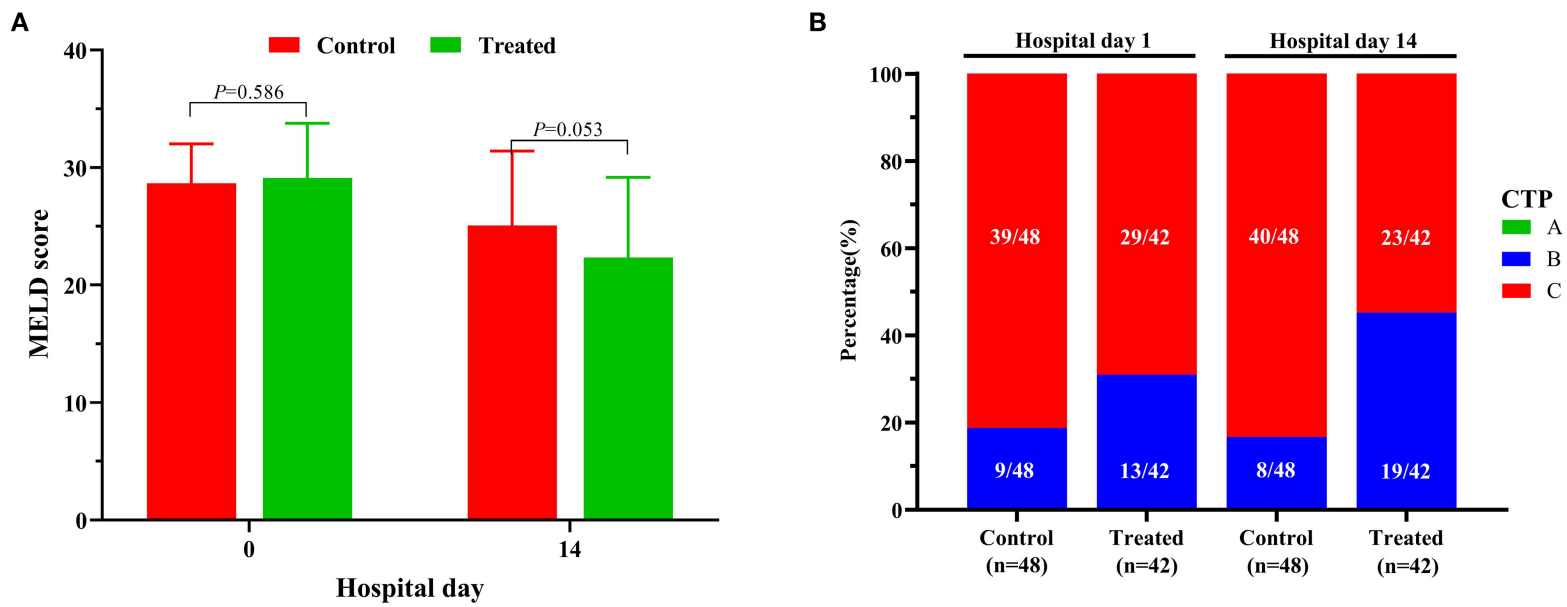
Changes of MELD **(A)** scores and CTP grades **(B)** between the treatment group and the control group.

During the 2-week observation period, the serum TBIL, DBIL, TBA, GGT and ALP levels of the two groups were significantly lower than those before treatment, which showed gradual downward trends (Figure 2). After 1 week of treatment, there was no significant difference in serum levels of TBil [304.5 (284.0–344.0]μmol/L vs. 324.0 (295.0–374.0) μmol/L, *P* = 0.071], DBil [222.5 (195.0–277.0) μmol/L vs. 253.0 (216.0–285.5) μmol/L, *P* = 0.099], TBA [178 (144–244) μmol/L vs. 211.0 (166.5–256.5) μmol/L, *P* = 0.072], and ALP [153 (134–177) IU/L vs. 174.5 (142.5–204.5) IU/L, *P* = 0.085] between the treatment group and control group; while serum GGT was significantly lower in control group than in treatment group [247.5 (188–301) IU/L vs. 220.0 (192.0–262.5) IU/L, *P* = 0.047]. However, after 2 weeks of treatment, the serum levels of DBil [153.5 (110.0–187.0) IU/L vs. 200.0 (166.0–241.5) IU/L, *P* = 0.008] and TBA [113.5 (71.0–148.0) μmol/L vs. 155.5 (123.5–195.0) μmol/L, *P* = 0.006] in the treatment group were significantly lower than those in the control group, although there was no significant difference in the distribution of serum TBIL, GGT and ALP levels. At the second week of treatment, the PT of the treatment group was lower than that of the control group [20.3 (18.3–22.5) second vs. 22.7 (20.5–31.1) seconds, *P* = 0.007] (Figure 3A), but there was no significant difference in serum albumin and creatinine between the two groups (Figures 3B,C). Although there was no significant difference in MELD score between two groups after 2 weeks of treatment (Figure 4A), more patients in the treatment group changed from CTP C-grade to B-grade than the control group (Figure 4B), and the difference was statistically significant (*P* = 0.003).

In addition, after 2 weeks of treatment, patients in the treatment group had lower blood ammonia levels than patients in the control group [44.5 (40.1–58.2) vs. 54.2 (49.6–71.9), *P* < 0.001]. After follow-up, it was found that the average length of hospital stay of the treatment group was significantly shorter than that of the control group [15.0 (13.0–17.0) vs. 21.5 (19.0–25.5), *P* < 0.001], although there was no significant difference in the short-term mortality between treatment group and control group [7.1% (3/42) vs. 10.4% (5/48) for 28-day mortality, *P* = 0.719; 9.5% (4/42) vs. 14.6% (7/48) for 90-day mortality, *P* = 0.465].

**Figure 5:**
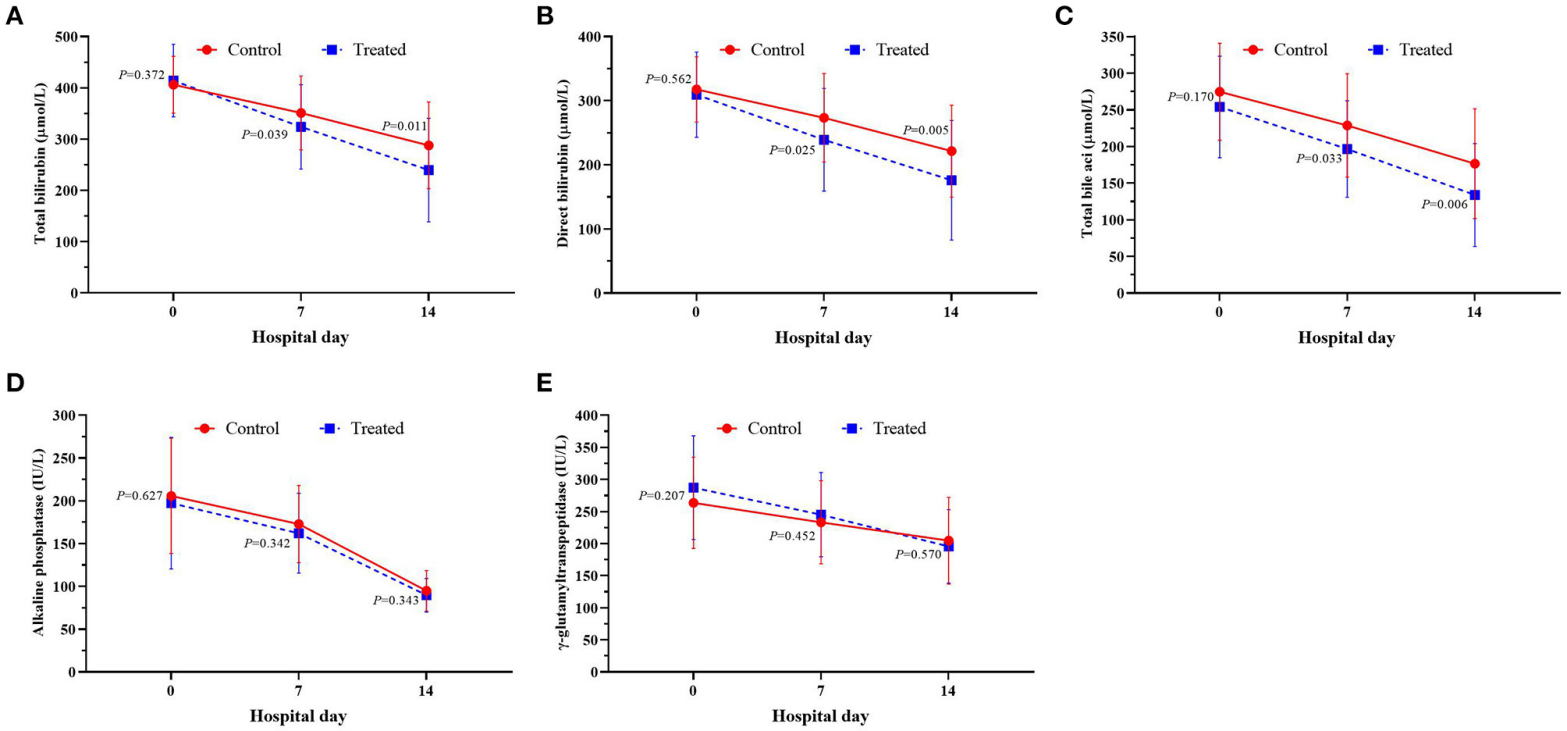
Changes of serum serum TBil **(A)**, DBil **(B)**, TBA **(C)**, ALP **(D)** and GGT **(E)** between the treatment group and the control group.

Subgroup analysis of HBV-ACLF patients with CTP C-grade showed that serum TBIL, DBIL and TB were all lower in the treatment group than those in the control group no matter in the first week or the second week after treatment, although there was no significant difference in the distribution of serum GGT and ALP between the two groups (Figure 5).

In the treatment group, there were 8 (19.2%) cases of chest tightness, 9 (21.4%) cases of nausea and 4 (9.5%) cases of vomiting; while 10 (23.8%) patients had vascular pain on the infusion side during the first infusion of NAC, and 6 of them had transient local skin swelling. None of the patients stopped using NAC because of the possible adverse reactions mentioned above. In the control group, 4 (8.3%) patients had chest tightness, 3 (6.3%) patients had nausea and 2 (4.2%) patients had vomiting.

## Discussion

HBV-ACLF is one of the common types of liver failure in China. At present, the pathogenesis of HBV-ACLF has not been fully elucidated (3). In this kind of patients, a large number of liver parenchymal cells necrosis can lead to a significant decline in liver metabolic detoxification function, and then lead to a large dose of endotoxin into the blood without inactivation (18). Endotoxemia is an important pathogenic factor that causes or aggravates severe liver damage (19). The increase of serum endotoxin level can stimulate the synthesis and expression of pro-inflammatory cytokines, aggravate the damage of liver function, and finally form a vicious circle (20). At present, it is believed that the activation of inducible nitric oxide synthase (iNOS) and its catalytic production of nitric oxide (NO) play an important role in LPS induced hepatocyte damage in patients with HBV-ACLF (18, 20).

Previous studies have reported that the iNOS is mainly distributed in hepatocytes and Kupffer cells in the liver (21, 22). It can be induced and activated by LPS and a variety of cytokines, and then produce a large number of endogenous NO with cytotoxic effect (21). As a free radical, NO can react with superoxide anion to form peroxynitrite, which has strong oxidation ability to protein, lipid and DNA, and then participates in the pathological process of hepatocyte injury. In human body, the serious damage of hepatocytes not only shows the release of ALT caused by the damage of hepatocyte membrane, but also leads to the blocked secretion and excretion of bile acid and bilirubin, and even affects the important synthesis function of liver. Thus, blocking or inhibiting the inflammatory response mediated by iNOS may help to alleviate or reverse the occurrence and development of severe liver injury (21, 23).

As we all know, acetylcysteine is the precursor of reduced glutathione (GSH), which is an oxygen free radical scavenger *in vivo*. The mechanism of its hepatoprotective effect is not very clear. At present, it is believed that NAC can not only directly react with ROS intermediate to inactivate and maintain the integrity of antioxidant enzyme system structure and functional recovery, but also increase the concentration of intracellular GSH and induce the accumulation of GSH into cells through deacetylation, so as to promote anti-oxidation, improve the ability of intracellular detoxification and reduce the release of oxygen free radicals (24). In addition, NAC may also play a protective role in liver by improving hemodynamics and oxygen transport capacity and expanding microcirculation (12, 14). At present, NAC is well-established in the treatment of acetaminophen induced fulminant liver failure, but its efficacy and mechanism in therapy of other forms of liver failure is unclear (16). In this study, we analyzed the efficacy and safety of adding NAC to the existing standard treatment for HBV-ACLF. Our results show that increasing NAC treatment can promote the remission of intrahepatic cholestasis and the improvement of coagulation function; and for CTP C-grade patients, increasing NAC treatment can make their liver function recovery better. At present, some scholars believe that NAC, as an antioxidant, can inhibit the activation of iNOS, and its role in alleviating hepatocyte injury and improving tissue hypoxia may be realized by regulating the redox state of hepatocytes and vasodilation (25). It has also been reported recently that NAC application could alleviate macrophages aggregation and inflammatory response, and mitigating liver injury and cell apoptosis (26).

In fact, NAC is also mentioned in AASLD guidelines for the treatment of liver failure caused by HBV infection (27). Unfortunately, in the past, because of the side effects of NAC (28), it was not routinely used in the treatment of HBV-ACLF in China. However, with the deepening of NAC research and improvement of its preparation process, the probability of side effects of NAC in clinical application is significantly reduced, so NAC is expected to be used in the comprehensive treatment of HBV-ACLF. In this study, although some patients reported chest tightness, nausea and vomiting, most of these adverse reactions were transient. After symptomatic treatment, the discomfort of the vast majority of patients was quickly relieved or disappeared, and no patients stopped treatment because of these possible related adverse reactions. Our preliminary results have suggested that the current clinical use of NAC has good safety. In this study, the temporary local skin swelling at the infusion site may be related to the excessive speed of intravenous infusion. If conditions permit, it is recommended to use infusion pump for intravenous infusion of NAC, which may help to reduce the probability of this potential adverse reaction.

There are some limitations in this study. In addition to the small sample size and retrospective study design, it is impossible to know whether these patients with HBV-ACLF have endotoxemia and whether they have iNOS and ROS mediated inflammatory disorder. The latter should be helpful to explain the good clinical efficacy of NAC. Therefore, it is necessary to investigate the mechanism of NAC in the treatment of HBV-ACLF in addition to a larger sample of prospective, randomized controlled studies. For a retrospective study, propensity score method is usually used for matching control group. However, due to the sample size, we did not use this routine protocol, so there is a potential bias in the grouping of patients.

## Data Availability Statement

The raw data supporting the conclusions of this article will be made available by the authors, without undue reservation.

## Ethics Statement

The studies involving human participants were reviewed and approved by the ethics committee of West China Hospital of Sichuan University. Written informed consent for participation was not required for this study in accordance with the national legislation and the institutional requirements.

## Author Contributions

M-LW, X-JY, X-LL, F-DW, JZ, Y-CT, Y-HW, and D-BW participated in the collection and analysis of data and the writing of the first draft of the article. E-QC designed the study, conducted the study supervision, and revised the manuscript. All authors contributed to the article and approved the submitted version.

## Conflict of Interest

The authors declare that the research was conducted in the absence of any commercial or financial relationships that could be construed as a potential conflict of interest.

## Publisher's Note

All claims expressed in this article are solely those of the authors and do not necessarily represent those of their affiliated organizations, or those of the publisher, the editors and the reviewers. Any product that may be evaluated in this article, or claim that may be made by its manufacturer, is not guaranteed or endorsed by the publisher.
